# Effect of inulin on small extracellular vesicles microRNAs in milk from dairy cows with subclinical mastitis

**DOI:** 10.1093/jas/skae366

**Published:** 2024-12-04

**Authors:** Wanjie Yu, Xuemei Nan, Martine Schroyen, Yue Wang, Mengting Zhou, Xiangfang Tang, Benhai Xiong

**Affiliations:** Precision Livestock and Nutrition Laboratory, Teaching and Research Centre (TERRA), Gembloux Agro-Bio Tech, University of Liège, Gembloux 5030, Belgium; State Key Laboratory of Animal Nutrition, Institute of Animal Science, Chinese Academy of Agricultural Sciences, Beijing 100193, P. R. China; Precision Livestock and Nutrition Laboratory, Teaching and Research Centre (TERRA), Gembloux Agro-Bio Tech, University of Liège, Gembloux 5030, Belgium; State Key Laboratory of Animal Nutrition, Institute of Animal Science, Chinese Academy of Agricultural Sciences, Beijing 100193, P. R. China; State Key Laboratory of Animal Nutrition, Institute of Animal Science, Chinese Academy of Agricultural Sciences, Beijing 100193, P. R. China; State Key Laboratory of Animal Nutrition, Institute of Animal Science, Chinese Academy of Agricultural Sciences, Beijing 100193, P. R. China; State Key Laboratory of Animal Nutrition, Institute of Animal Science, Chinese Academy of Agricultural Sciences, Beijing 100193, P. R. China

**Keywords:** inulin, inflammation, milk microRNA, pathways, subclinical mastitis

## Abstract

Milk contains microRNAs (miRNA) that are shielded by small extracellular vesicles (sEVs). Beyond variations among individuals, many factors including nutrition play a role in shaping miRNA expression profiles. This study is to explore milk-derived sEVs-miRNA variations induced by inulin supplementation in subclinical mastitis-suffering cows. Fourteen lactating cows diagnosed with subclinical mastitis were equally assigned to either an inulin or a control group. Apart from total mixed rations, cows in the inulin group were provided with 300 g/d inulin during the morning feeding, while the control group did not receive any supplement. Following 1 wk of adaptation and 5 wk of treatment, sEVs-miRNA were isolated from the milk of each cow. RNA is subjected to high-throughput sequencing and differentially expressed (DE) miRNA (*P* < 0.05 and ∣ log2FC∣> 1) were detected through bioinformatics analysis. Gene Ontology (GO) enrichment and Kyoto Encyclopedia of Genes and Genomes (KEGG) pathway analyses were conducted to examine the target genes of DE miRNA. A sum of 350 miRNA was discovered, including 332 in the control group and 249 in the inulin group. Among these, 9 miRNA showed differential expression within the 2 groups, including 3 upregulated and 6 downregulated in the inulin group. The DE miRNA participates in regulating organismal systems, cellular processes, and signal transduction, which may affect inflammatory response and milk production. Overall, our study provides insight into the micromolecular-level mechanism of inulin in alleviating subclinical mastitis in dairy cows.

## Teaser

Inulin supplements can influence inflammatory response and milk production by affecting milk-derived small extracellular vesicle microRNA expression.

## Introduction

Milk contains abundant nutrients and bioactive substances, providing a comprehensive source of essential nutrients and immune protection for newborns and juvenile animals. Bioactive substances in milk have shown regulatory potential in immunity, infection, and inflammation control (Hill and Newburg, 2015). In recent decades, extracellular vesicles (**EVs**), particularly small extracellular vesicles (**sEVs**) with a size range of 30 to 150 nm, have been widely studied as bioactive compounds for their biological functions (Gao et al., 2021).

Exogenous EVs have a double-layer structure and are resistant to acidic conditions and RNases (Izumi et al., 2015). They protect their cargo from harsh conditions, allowing passage through the gastrointestinal tract and absorption in the small intestine (Stoorvogel et al., 2002). These EVs can deliver microRNAs (**miRNA**) to target cells, thus modulating the target cell by regulating biochemical pathways (Ong et al., 2021). A study examining miRNA profiles prior to and after a *Staphylococcus aureus* infection in cattle identified 22 mammary-expressed objective genes from the revealed differentially expressed (**DE**) miRNA. These objective genes participate in modulating immune processes and inflammation responses (Sun et al., 2015). Milk-derived sEVs can also regulate tumor occurrence and transition, mediating apoptosis, and inflammation (Samuel et al., 2017; El-kattawy et al., 2021). Bovine milk-derived sEVs have revealed protective effects from oxidative stress, affected metabolism processes, and improved energy status (Chen et al., 2020; Wang et al., 2021a). Additionally, they were shown to alter microbial communities in mice through milk intake containing sEVs (Zhou et al., 2019). Conversely, EVs can also have negative effects in certain situations. For instance, despite reducing tumor growth, milk EVs can also accelerate cancer metastasis by inducing epithelial-to-mesenchymal transition in cancer cells (Samuel et al., 2021).

As the key cargo of EVs, miRNA modulates gene expression at the post-transcriptional level by base-pairing with complementary messenger RNA targets, influencing protein synthesis (Kuokkanen et al., 2010; Ong et al., 2021). They play vital roles in biological processes, cellular components, and molecular functions by modifying gene expression (Zhang et al., 2022b) and influencing numerous pathways, including signal transduction, diseases, and cellular processes (Quan et al., 2020). The miRNA expressions vary widely in different studies, influenced by factors such as breed, environment, and lactation period (Fan et al., 2021a; Yun et al., 2021). Furthermore, miRNAs are significantly altered during different lactation periods, abundant miRNAs regulate basic metabolic, cellular, and immunological functions (Do et al., 2017). And 7 milk-specific miRNA consistently expressed, offering the potential for quality control (Chen et al., 2010).

Moreover, variations in EVs-miRNA may reflect the health conditions of animals. Abnormal miRNA expression can contribute to disease pathogenesis (Do et al., 2021). The expression levels of miR-142-5p and miR-223 were increased after Streptococcus uberis infection (Sun et al., 2015). Similarly, lipoteichoic acid can activate inflammatory responses and increase miR-23a targeting of PI3K to regulate inflammation (Cai et al., 2021). Uterine infections in dairy cows result in 30 DE miRNA compared to healthy cows. These miRNAs play a crucial role in combating pathogen infestation and regulating inflammation (Kasimanickam and Kastelic, 2016). The above studies suggest the capability of miRNA profiles as disease biomarkers and metabolic condition detection (Colitti et al., 2020). Different feed compositions also lead to variations in miRNA expression. Feed restrictions affected the abundance of miRNA in milk EVs (Leduc et al., 2023). Similarly, substituting a portion of alfalfa hay with a mixture of byproducts in cattle feed resulted in 9 DE miRNA in milk (Quan et al., 2020). These miRNAs are shown to participate in processes such as fat metabolism, taurine and hypotaurine metabolism, and glycosphingolipid biosynthesis. The diversity of miRNA composition and expression under various nutritional and health conditions warrants further attention.

Subclinical mastitis is an inflammatory reaction triggered by pathogenic bacteria in dairy udders without evident signs (Ruegg, 2017). It decreases the milk quality and milk yield, affecting the profit of dairy farms (Ruegg, 2017). The health conditions of the mammary gland affect miRNA profiles, leading to DE miRNA between healthy and subclinical cows (Stefanon et al., 2023).

Plant components are widely used in preventing and treating inflammations to reduce the use of antibiotics during farming (Khan et al., 2023). Inulin is often utilized for its prebiotic properties, given its capability to promote the growth of microorganisms in the hindgut (Usman et al., 2021). Additionally, it can promote the health condition of the animal. Studies have shown that both antioxidant and immune functions are enhanced in cows when their feed is supplemented with inulin (Zhao et al., 2022). Similarly, inulin supplementation may help mitigate subclinical mastitis by regulating microbial communities and metabolites within the mammary tissue (Wang et al., 2022). Moreover, supplementing with inulin has been shown to increase milk production and improve the milk fatty acid profile (Zhao et al., 2022). However, the molecular-level changes behind these results are unclear. We hypothesize that supplementing inulin in the diet could alter bovine milk-derived sEVs-miRNA profiles.

Based on a previous study, supplementation with inulin at 300 g/d showed the best results in reducing pathogenic bacteria in the mammary tissue (Wang et al., 2022). Therefore, we have chosen the same dose of inulin for this study. The health condition of the mammary tissue can be reflected in milk. Analyzing changes in milk-derived sEVs-miRNA profiles induced by inulin may help understand the mitigation process of inflammation due to inulin by identifying involved pathways. The objective of this study is to supply new evidence for differences in miRNA expression induced by inulin in subclinical mastitis-suffering cows.

## Materials and Methods

### Ethics Statement

The animal management, experimental procedures, animal welfare, and ethics protocol of this study were in compliance with the academy’s guidelines for animal research and approved by the Animal Ethics Committee of the Chinese Academy of Agricultural Sciences (Beijing, China; approval number IAS-2020-92).

### Animals, Diets, and Sample Collection

The current study was performed in July and August 2020 at a commercial dairy farm in Beijing, China. The selection of the animals was according to the outcomes of the California mastitis test (**CMT**), milk cell count data, and clinical features. Briefly, milk was mixed with an equal volume of reacting solution of the CMT, and 4 levels were graded according to milk color and form (Wang et al., 2020). The cows that tested positive or weakly positive in CMT and showed no diagnostic signs in their udders were defined as subclinical mastitis-suffering cows. Fourteen subclinical mastitis-suffering Holstein dairy cows (days in milk = 161.43 ± 28.11 d; parity = 2.86 ± 1.29; milk yield = 28.26 ± 5.36 kg/d; milk somatic cell counts = 6,591.64 ± 973.13 × 10^3^ cells/mL, Table 1) were divided into a control and an inulin group at random (*n* = 7 each). The feeding regimen remained consistent with our previous research (Yu et al., 2023b). Cows were individually housed and received total mixed rations (Supplemental Table S1) with or without 300 g/d of inulin supplement for the inulin and control group, respectively (Wang et al., 2022). Total mixed rations were provided at 08.00, 14.00, and 20.00 hours, with unrestricted access to water. Inulin with purity over 85% was supplemented in each cow in the inulin group through oral administration using a feeder (Boehringer-Ingelheim, Biblach, Germany) during morning feeding (Wang et al., 2022). The experiment constituted of a 1-wk adaptation phase followed by a 5-wk treatment phase.

**Table 1.** Basic information (Mean) on dairy cows with subclinical mastitis in control (*n* = 7) and inulin group (*n* = 7)

	Control group	Inulin group	Average	SEM^1^	*P* values
Days in milk	174.29	148.57	161.43	7.51	0.086
Parity	2.57	3.14	2.86	0.35	0.430
Milk yield, kg/d	30.73	25.80	28.26	1.43	0.085
Milk somatic cell counts (×10^3^ cells/mL)	6,412.71	6,770.57	6,591.64	260.08	0.513
CMT^2^ results	+/++^3^	+/++	/	/	/

^1^SEM, standard error of the mean.

^2^CMT, California mastitis test.

^3^+, weakly positive; ++, positive.

Milk samples were obtained from a bottle connected to the automatic milking system at 06.00, 12.00, and 18.00 hours on the last day of the experiment. The samples were mixed at a ratio of 4:3:3, and preserved at −80 °C for subsequent procedures.

### Isolation and characterization of milk small extracellular vesicles

Milk samples were defrosted in a room-temperature water bath. Five milliliters of milk sample was centrifuged for 10 min at 1,200 × *g* at 4 °C 2 times to eliminate fat and large debris. Collecting the clarified supernatant and centrifuged at 21,500 × *g* at 4 °C for 50 min to eliminate casein and remnant fat. The sEVs were isolated following the previously described protocol (Yu et al., 2023b). In summary, the supernatant was balanced to 15 mL by mixing with PBS, transferred into the pre-cleaning column, and centrifuged to eliminate the cellular debris. Nonspecific protein aggregates, lipoproteins, and cytokines were eliminated by repeatedly centrifuging the flow-through in an Amicon Ultra-15 filter. Then, an isolation column was used to isolate sEVs. Equilibration buffer (5 mL) was incorporated into the column and centrifuged at 3,000 × *g* for 2 min. After replacing the tube, the sample was loaded into the same column and centrifuged to make the sEVs bound with the membrane. The sEVs were washed out by the wash buffer. The ultimate eluate was obtained and preserved at −80 °C.

Nanoparticle tracking analysis was conducted using ZetaView (S/N 252, Particle Metrix, Germany) with a resolution of 0.703 µm/px, to identify the concentration and partical size of sEVs. The dilution factor was 500 with PBS.

### Small RNA analysis

#### RNA library construction and sequencing

The isolation of small RNA followed the protocol of miRNeasy Mini Kit (cat. No. 217004, QIAGEN, Germany). Concisely, sEVs samples were initially lysed and homogenized in a highly denaturing buffer, effectively deactivating RNases and ensuring the preservation of intact RNA during purification. By mixing with ethanol, the samples were loaded into the column. The contaminants were passed through the membrane and washed away. The eluted, high-quality RNA was acquired for subsequent examination.

The small RNA sequencing was executed by VivaCell (Shanghai, China) using the Illumina NovaSeq 6000, PE150. Briefly, T4 RNA Ligase connected 3ʹ and 5ʹ adapters to the ends of RNA molecules, and the library was reverse transcribed and then amplified. The purified and recovered cDNA constructs were subjected to library checking and normalization.

#### Bioinformatics analysis

The bioinformatics analysis was executed by VivaCell (Shanghai, China). In brief, raw data were converted from basic reads by base calling. Reads with 3ʹ adapters, without 5ʹ primer contaminants, and ranging between 15 nt and 41 nt were kept. The quality of the read was controlled by Fastx_toolkit (version 0.0.13; Gordon and Hannon, 2010). The clean reads were acquired by filtering reads containing N bases using Fastq (Patel and Jain, 2012). Non-coding RNAs, including long non-coding RNAs, other small RNAs, ribosomal RNAs, and small nucleolar RNAs, were annotated by searching through the Rfam (v.10.1) database using bowtie (Griffiths-Jones et al., 2003). The miRBase (v.21) database was used to recognize miRNA and investigate their expression modes (Kozomara et al., 2019). The miRDeep2 was used for quantitative statistical analysis of small RNAs and for predicting potential miRNA precursors and mature miRNA sequences (Friedländer et al., 2011). The analyses of Gene ontology (**GO**) enrichment and the Kyoto Encyclopedia of Genes and Genomes (**KEGG**) pathway were conducted according to the forecasted DE miRNA target genes.

#### Statistical analysis of data

Basic informations, annotated small RNAs, the length distribution of miRNA, and the number of known miRNA were analyzed for differences using a T-test with SPSS 26, with *P* < 0.05 considered as a significant difference. The DE miRNA were analyzed using the R packages “DESeq2” (Au—Liu et al., 2021) and selected based on *P* < 0.05 and ∣ log2FC∣> 1. The miRNA names were present as miRNA/precursor. The padj was obtained for multiple testing by adjusting *P*-value using the Benjamin-Hochberg correction (Tino, 2009). GO and KEGG analysis was carried out by hypergeometric distribution with R (version R3.5.1), and taking *P*adj < 0.05 as the significant enrichment standard.

## Results

### Confirmation of bovine milk small extracellular vesicles

**Figure 1.**
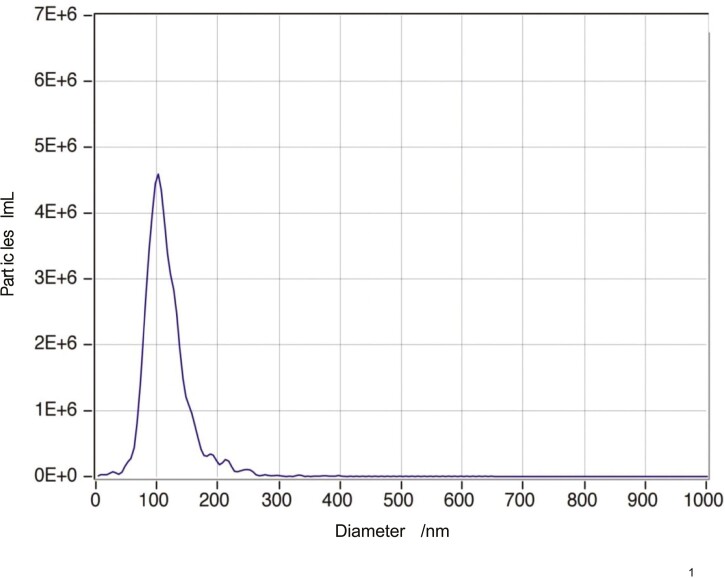
Particle size distribution of bovine milk-derived small extracellular vesicles from cows with subclinical mastitis.

The EVs particle size distribution was analyzed by nanoparticle tracking (Figure 1). The diameter of EVs reached its peak at 102.2 nm, with 97.7% of all particles falling within the range of 30 to 150 nm. particle diameters ranging from accounting for.

### Profiling of small extracellular vesicles-RNAs in bovine milk

**Figure 2.**
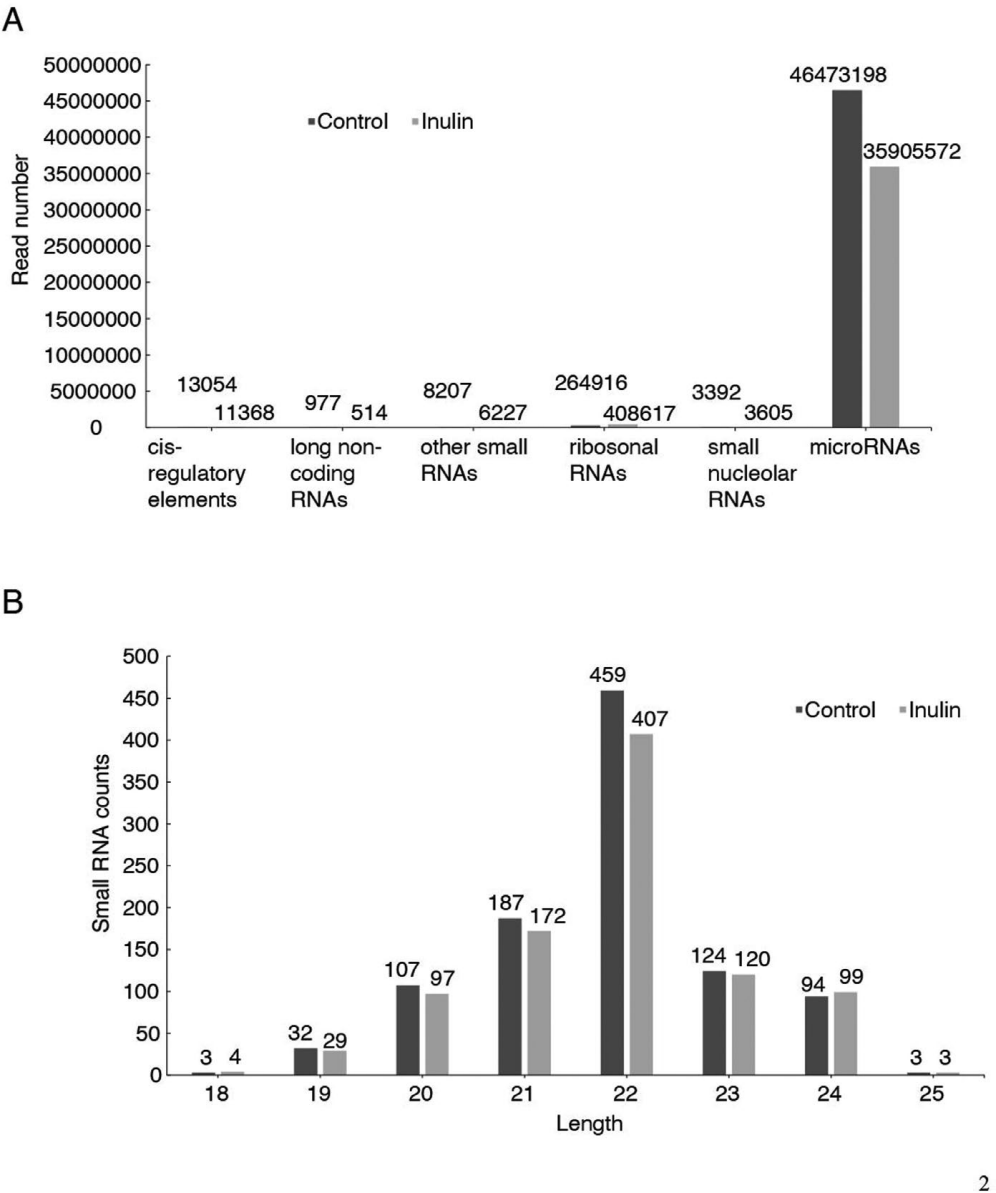
Profiling of annotated small RNAs and length distribution of microRNAs derived from milk small extracellular vesicles in dairy cows with subclinical mastitis in the control and inulin group. (A) Profiling of annotated small RNAs derived from milk small extracellular vesicles in dairy cows with subclinical mastitis in the control and inulin group. (B) The length distribution of microRNAs derived from milk small extracellular vesicles in dairy cows with subclinical mastitis in the control and inulin group.

The total numbers of annotated RNAs, including cis-regulatory elements, long non-coding RNAs, other small RNAs, ribosomal RNAs, small nucleolar RNAs, and miRNA, are shown in Figure 2A. The miRNA (Figure 2B) varied in length from 18 to 25 nt, and most were 22 nt long (45.49% and 43.72% for the control and inulin groups, respectively).

**Figure 3.**
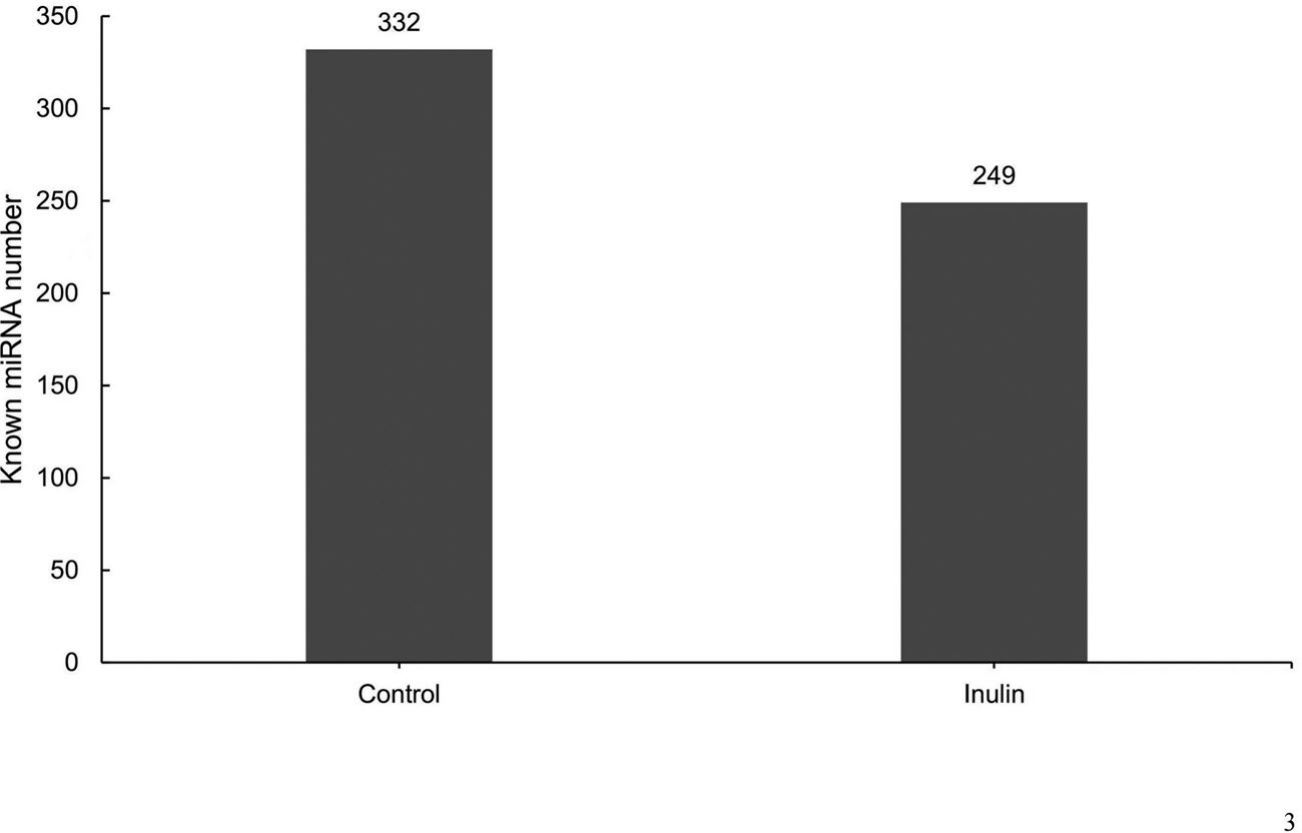
The number of known milk small extracellular vesicles derived microRNAs in the control and inulin group.

A combined total of 350 miRNAs were detected among all samples. There were 332 miRNA in the control group and 249 miRNA in the inulin group (Figure 3; Supplementary Table S2).

### Most abundant small extracellular vesicles-microRNAs in bovine milk

Table 2 displays the top 20 miRNA with the highest expression levels in both groups. Among them, 18 miRNA (let-7b/let-7b, let-7a-5p/let7a-1, let-7a-5p/let7a-2, let-7a-5p/let7a-3, miR-1246/mir-1246, miR-423-5p/mir-423, let-7f/let-7f-1, let-7f/let-7f-2, let-7c/let-7c, let-7g/let-7g, miR-16a/mir-16a, miR-26a/mir-26a-1, miR-26a/mir-26a-2, miR-320a/mir-320a-1, miR-320a/mir-320a-2, miR-200c/mir-200c, miR-191/mir-191, and miR-151-5p/mir-151) were expressed at high levels in both groups.

**Table 2.** Top 20 highly expressed small extracellular vesicles derived microRNAs in milk from dairy cows with subclinical mastitis in 2 groups

		Group			
		Control (*n* = 7)		Inulin (*n* = 7)	
microRNAs		Mean	Percentage, %	Mean	Percentage, %
1	let-7b/let-7b	17,004.86	14.95	24,012.14	19.34
2	let-7a-5p/let-7a-3	17,868.43	15.71	22,381.71	18.03
3	let-7a-5p/let-7a-1	17,862.43	15.70	22,384.71	18.03
4	let-7a-5p/let-7a-2	17,859.71	15.70	22,377.43	18.02
5	miR-1246/mir-1246	12,212.71	10.74	6,965.57	5.61
6	miR-423-5p/mir-423	7,105.71	6.25	3,811.00	3.07
7	let-7f/let-7f-2	3,107.14	2.73	2,894.43	2.33
8	let-7f/let-7f-1	3,011.57	2.65	2,801.57	2.26
9	let-7c/let-7c	2,080.14	1.83	3,360.86	2.71
10	let-7g/let-7g	1,283.86	1.13	1,037.71	0.84
11	miR-16a/mir16a	1,366.14	1.20	785.71	0.63
12	miR-26a/mir26a-2	1,366.14	1.20	779.00	0.63
13	miR-26a/mir-26a-1	1,365.71	1.20	778.86	0.63
14	miR-320a/mir-320a-1	819.57	0.72	869.43	0.70
15	miR-320a/mir-320a-2	819.57	0.72	869.43	0.70
16	miR-200c/mir-200c	858.43	0.75	760.57	0.61
17	miR-191/mir-191	821.57	0.72	355.14	0.29
18	miR-151-5p/mir-151	439.14	0.39	706.14	0.57
19	miR-30a-5p/mir-30a	583.71	0.51	316.43	0.25
20	let-7i/let-7i	384.29	0.34	380.00	0.31
	Total		95.13		95.55

### DE small extracellular vesicles-microRNAs in bovine milk

**Figure 4.**
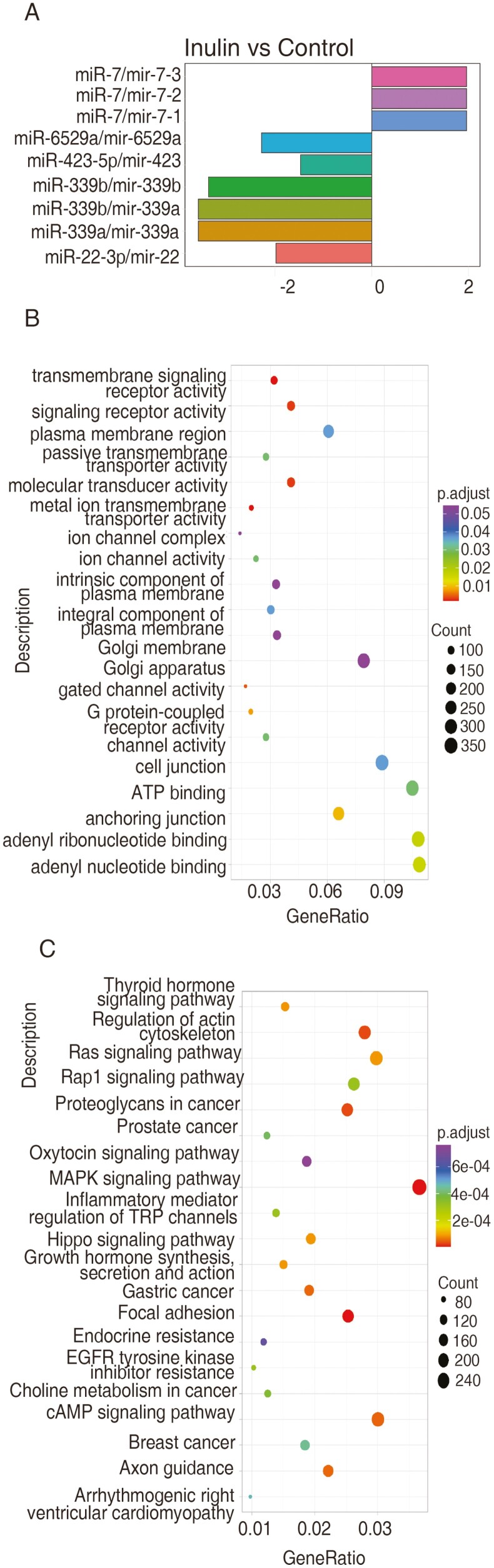
Differentially expressed milk-derived small extracellular vesicle microRNAs and functional analysis of their target genes in cows with subclinical mastitis between the control and inulin group. (A) Differentially expressed milk-derived small extracellular vesicle microRNAs from cows with subclinical mastitis between the control and inulin groups. (B) Gene ontology enrichment analysis for the top 20 functions of target genes related to differentially expressed milk-derived small extracellular vesicle microRNAs from cows with subclinical mastitis. (C) Kyoto Encyclopedia of Genes and Genomes analysis for the top 20 pathways influenced by target genes from differentially expressed milk-derived small extracellular vesicle microRNAs in cows with subclinical mastitis.

Between the 2 groups, there were 9 miRNA that demonstrated differential expression. Compared to control group, miR-7/mir-7-3, miR-7/mir-7-2, and miR-7/mir-7-1 were upregulated, while miR-6529a/mir-6529a, miR-423-5p/mir-423, miR-339b/mir-339a, miR-339b/mir-339b, miR-339a/mir-339a, and miR-22-3p/mir-22 were downregulated in the inulin group (Figure 4A). One of the DE miRNAs (miR-423-5p) was also found to be highly expressed.

The predicted target genes of DE miRNA were applied for a GO enrichment analysis. The top 20 functional categories are shown in Figure 4B. A total of 16 target genes were significantly enriched in the categories of molecular function and cellular component. In the KEGG pathway analysis, 111 target genes were significantly enriched, with the top 20 pathways displayed in Figure 4C. These include organismal systems (sensory system, endocrine system, development and regeneration), cellular processes (cell motility, cellular community), signal transduction, and human diseases (cancer, drug resistance, cardiovascular disease).

## Discussion

Daily milk production and its fluctuations are indicators of the health of dairy cows (Kok et al., 2021). Mastitis, including subclinical mastitis, is mammary inflammation in lactating cows caused by bacterial infections and physical factors such as excessive milking settings and improper operation (Cai et al., 2021; Gasparik et al., 2022). Subclinical mastitis, like clinical mastitis, negatively impacts animal welfare and the farm economy but is more challenging to detect due to the absence of significant changes in milk and clear clinical signs in the udder (Cai et al., 2021; Ibrahim et al., 2023).

Currently, antimicrobials are widely employed as an effective treatment for mastitis (Smulski et al., 2020). However, their overuse has led to antibiotic resistance, reducing drug efficacy. It is often unnecessary to treat non-severe mastitis in dairy cows (Nobrega et al., 2020). With the prohibition of feed antibiotics and a trend toward reducing antimicrobials in farming, plant extracts are gathering considerable interest in contemporary research. These extracts contain various bioactive components, including polyphenols, polysaccharides, and essential oils. Numerous studies have focused on their potential for disease prevention and antibacterial activity against multiple microorganisms. Several plant compounds have been found to have antimicrobial potential and to improve antioxidant and immune functions, suggesting that plant extracts can reduce inflammation, enhance production performance, and benefit animal health (Blaxland et al., 2022; Cao et al., 2023; Guo et al., 2023). Furthermore, plant extracts can impact the microbiota structure (Su et al., 2023; Yu et al., 2023a).

Inulin, a plant extract, is often used as a prebiotic supplement to modulate digestive tract microbiota (Zhu et al., 2020; Wang et al., 2021b). Inulin also increases milk production, reduces inflammatory factors, and alleviates bovine mastitis symptoms (Wang et al., 2021b; Zhao et al., 2022). Recently, inulin has been used in bovine mastitis therapy. To understand its molecular impact on bovine mastitis, in this study, we monitored differences in mastitis mitigation and investigated inulin’s anti-inflammatory mechanism.

The EVs are small nanovesicles, classified into sEVs and ectosomes, based on partical size, cargo sorting, and biogenesis (Gao et al., 2021). According to nanoparticle tracking analysis, 97.7% of milk-derived particles are sEVs. This study identified 350 miRNAs, with 332 in the control group and 249 in the inulin group. These numbers align with previous findings by Tsukada et al. (Tsukada et al.) who found 277 miRNA in milk-derived sEVs from healthy cows (Tsukada et al., 2022), along with the findings of Quan et al. (2020) who illustrated the presence of 276 miRNA in milk-derived EVs (Quan et al., 2020). We found that inulin supplementation did not significantly affect miRNA length, known miRNA numbers, or small RNA read numbers (including cis-regulatory elements, long non-coding RNAs, other small RNA, ribosomal RNAs, small nucleolar RNAs, and miRNA). Nineteen out of the top 20 miRNA with high expression levels in this study, excluding miR-1246, were earlier reported in milk EVs from healthy cattle (Quan et al., 2020; Li et al., 2022). These miRNAs may influence milk’s basic functions in growth, development, and immunity. For instance, the let-7 family, including let-7a-5p, let-7b, let-7c, let-7f, and let-7g in this study, is known for regulating cell proliferation, differentiation, apoptosis, invasion, and migration (Liu and Jiang, 2020; Egea et al., 2021). These miRNAs likely contribute to regulating breast tissue growth and lactation process (Quan et al., 2020). Besides, miR-16a can target large tumor suppressor kinase 1 and has been demonstrated to manipulate milk lipid metabolism in the epithelial cells of bovine breast tissue (Chen et al., 2019). In addition, miR-423-5p, miR-26a, let-7a-5p, let-7c, and let-7f may modulate inflammation (Kreuzer-Redmer et al., 2016; Zhang et al., 2021; Yu et al., 2022). These miRNAs highlight the fundamental mechanism of the immune response in milk. The highly expressed miR-1246 was found upregulated in cows suffering from mastitis (Lai et al., 2020) or those challenged with LPS (Sullivan et al., 2022). Although we found no significant changes in miR-1246 between the 2 groups, the normalized mean reads decreased numerically from 12,212.71 to 6,965.57, suggesting the potential alleviation of mastitis.

Individual differences are one of the factors that induce variations in miRNA profiles. Dairy cows show distinct levels of immunoglobulin and specific antibody concentrations in milk, which reflects their diverse immune response levels (Ross et al., 2021). The current study demonstrated significant individual differences within each group due to the animals’ diverse backgrounds. Furthermore, the variations between the control and inulin groups might be associated with changes in nutritional factors. Numerous studies have confirmed that plant miRNA can escape from the digestive tract, be assimilated in the small intestine, and influence disease progression by regulating miRNA expression in multiple animal species (Saiyed et al., 2022). Moreover, inulin, as a prebiotic, not only alters the microbiota profile in the gastrointestinal tract but can also modulate the microbiota and metabolite levels in mammary organs (Wang et al., 2022). Therefore, consuming inulin may affect the miRNA profile directly through cell communication or indirectly by modulating the microbiota composition. Among the DE miRNA, the upregulated miR-7 is known to target FAK through the ERK/MAPK signaling pathway which suppresses cellular activities (Cao et al., 2016). The downregulation of miR-6529a promotes preadipocyte multiplication, differentiation, and lipid deposition (Ran et al., 2022). This lipid deposition also explains the suppression of lipid metabolism in another inulin supplement study (Wang et al., 2021b). The inhibition of miR-423-5p targets GRIM-19 and suppresses prostate cancer (Lin et al., 2019). miR-339 and miR-22, can regulate cell motility, inflammation, and apoptosis, suggesting their roles in alleviating mastitis (Xie et al., 2020; Fan et al., 2021b).

The GO enrichment analysis revealed that DE miRNA target genes affect key (top 20) molecular functions and cellular components. They primarily participate in maintaining cell and tissue structure and function, as well as in signal transduction. The KEGG analysis of DE miRNA target genes suggests that inulin may regulate signaling pathways, including MAPK, cAMP, Ras, and Rap1. These signaling pathways influence cellular processes such as cell proliferation, migration, apoptosis, etc (Olson and Marais, 2000). The activation of the MAPK pathway can accelerate the inflammation process by stimulating the secretion of inflammatory factors (Zhang et al., 2022a). Rap1 can regulate MAPK activity and plays a role in immune regulation (Minato et al., 2007). In a like manner, another study found that inulin acts as a potent modulator of inflammation through MAPK activation (Wu et al., 2017). The activation of TRP channels influences the MAPK signaling pathway, consequently inducing an inflammatory response (Zhang et al., 2023). Moreover, thyroid hormone regulates growth, development, and metabolism, and is essential for maintaining immune function and response (Lasa and Contreras-Jurado, 2022). Additionally, DE miRNA target genes may influence milk production. The cAMP signaling pathway participates in the regulation of lipolysis by influencing protein expression levels (Ikoma-Seki et al., 2015). The growth hormone and oxytocin signaling pathways stimulate milk secretion and influence milk composition (Sadiq and Tadi, 2024). These findings may explain the effect of inulin on mitigating inflammation and improving milk production in cows with mastitis.

The DE miRNA identified in the current study provides evidence of inulin’s potential to reduce bovine subclinical mastitis at the molecular level. Due to the fact that inulin can directly modulate host mucosal signaling to influence response to bacterial infection (Wu et al., 2017), further in vitro studies could determine whether inulin supplements affect miRNA expression straightforwardly or through intermediate products or compounds generated by the supplement within the animal body.

## Conclusion

Inulin supplementation impacts the expression of bovine milk sEVs-miRNA and alters the highly expressed miRNA in cows with subclinical mastitis. The target genes of these DE miRNAs influence organismal systems, cellular processes, and signal transduction, which may affect inflammatory response and milk production. Several studies have shown that milk-derived EVs-miRNA plays regulatory roles in consumers and even their offspring. Thus, inulin may improve milk quality by altering bioactive components in the milk.

## Supplementary Material

skae366_suppl_Supplementary_Table_S1

skae366_suppl_Supplementary_Table_S2

## Abbreviations

CMT: California mastitis test
DE: differentially expressed
EVs: extracellular vesicles
GO: Gene Ontology
KEGG: Kyoto Encyclopedia of Genes and Genomes
miRNA: microRNAs
sEVs: small extracellular vesicles

## Data and Availability

The raw data files were uploaded into the NCBI GEO database (https://www.ncbi.nlm.nih.gov/ accession number: GSE256521).
