# Supplementary material for: Effect of inulin on small extracellular vesicles microRNAs in milk from dairy cows with subclinical mastitis
Source: J Anim Sci. 2024 Dec 4;101:skae366. doi: 10.1093/jas/skae366 (PMC11664108; doi:10.1093/jas/skae366)
Supplement: skae366_suppl_Supplementary_Table_S1 [file skae366_suppl_supplementary_table_s1.docx]

**Supplemental Table S1**

TMR^1^ ingredients and nutrient components for dairy cows suffering from sub-clinical mastitis in both control and inulin groups

| Ingredient, % of DM | Content |
| --- | --- |
| Corn silage | 48.27 |
| Steam-flaked corn | 10.45 |
| Alfalfa hay | 8.18 |
| Cottonseed | 7.36 |
| Oat grass | 3.18 |
| Extruded soybean | 0.45 |
| Sprayed corn husk | 2.27 |
| Megalac^2^ | 0.45 |
| Fatty powder | 0.91 |
| Beet granules | 1.36 |
| Rapeseed meal | 2.20 |
| Hulless oat straw | 2.30 |
| Sunflower meal | 0.64 |
| 5% Premix^3^ | 4.91 |
| DDGS | 4.42 |
| NaHCO3 | 1.50 |
| MgO | 0.80 |
| Sodium diacetate | 0.33 |
| Nutrient composition, % of DM |  |
| CP | 16.91 |
| NDF | 30.09 |
| ADF | 17.32 |
| EE | 4.93 |
| Ca | 0.81 |
| P | 0.48 |
| NEL, Mcal/kg | 1.72 |

^1^Abbreviations: TMR = total mixed rations; DDGS = distiller’s dried grain with soluble; EE = ether extract; NEL = net energy for lactation.

^2^Megalac = a complex fatty acid calcium (Jianhe Animal Husbandry Co., Ltd., Shanghai, China).

^3^5% Premix: including (per kg of DM) 400 000 IU of vitamin A, 320 000 IU of vitamin D3, 1200 IU of vitamin E, 1 400 mg of Cu, 12 000 mg of Zn, 60 000 mg of Fe, 12 000 mg of Mn, 40 mg of Se, 400 mg of I, 160 mg of Co, 28% of Ca and 5.4% of P.
