# Supplementary material for: Effect of inulin on small extracellular vesicles microRNAs in milk from dairy cows with subclinical mastitis
Source: J Anim Sci. 2024 Dec 4;101:skae366. doi: 10.1093/jas/skae366 (PMC11664108; doi:10.1093/jas/skae366)
Supplement: skae366_suppl_Supplementary_Table_S2 [file skae366_suppl_supplementary_table_s2.docx]

**Supplemental Table S2**

The known milk small extracellular vesicles derived microRNAs in the control and inulin group.

|  | | Group | | |
| --- | --- | --- | --- | --- |
|  | | Control (n = 7) | | Inulin (n = 7) |
| microRNAs | | Mean | | Mean |
|  | bta-let-7b | | 17004.86 | 24012.14 |
|  | bta-let-7a-5p | | 17868.43 | 22381.71 |
|  | bta-let-7a-5p | | 17862.43 | 22384.71 |
|  | bta-let-7a-5p | | 17859.71 | 22377.43 |
|  | bta-miR-1246 | | 12212.71 | 6965.57 |
|  | bta-miR-423-5p | | 7105.71 | 3811.00 |
|  | bta-let-7f | | 3107.14 | 2894.43 |
|  | bta-let-7f | | 3011.57 | 2801.57 |
|  | bta-let-7c | | 2080.14 | 3360.86 |
|  | bta-let-7g | | 1283.86 | 1037.71 |
|  | bta-miR-16a | | 1366.14 | 785.71 |
|  | bta-miR-26a | | 1366.14 | 779.00 |
|  | bta-miR-26a | | 1365.71 | 778.86 |
|  | bta-miR-320a | | 819.57 | 869.43 |
|  | bta-miR-320a | | 819.57 | 869.43 |
|  | bta-miR-200c | | 858.43 | 760.57 |
|  | bta-miR-191 | | 821.57 | 355.14 |
|  | bta-miR-151-5p | | 439.14 | 706.14 |
|  | bta-miR-30a-5p | | 583.71 | 316.43 |
|  | bta-let-7i | | 384.29 | 380.00 |
|  | bta-miR-30d | | 386.71 | 267.71 |
|  | bta-let-7d | | 279.14 | 203.57 |
|  | bta-miR-7 | | 135.86 | 332.43 |
|  | bta-miR-7 | | 135.86 | 332.29 |
|  | bta-miR-7 | | 135.86 | 332.14 |
|  | bta-miR-375 | | 173.57 | 290.86 |
|  | bta-let-7e | | 189.14 | 227.14 |
|  | bta-miR-2904 | | 136.86 | 279.43 |
|  | bta-miR-2904 | | 136.86 | 279.43 |
|  | bta-miR-2904 | | 136.86 | 279.43 |
|  | bta-miR-26b | | 230.29 | 173.57 |
|  | bta-miR-11980 | | 89.14 | 297.43 |
|  | bta-miR-184 | | 122.29 | 160.43 |
|  | bta-miR-98 | | 128.00 | 145.00 |
|  | bta-miR-2284x | | 176.86 | 90.86 |
|  | bta-miR-2284y | | 176.86 | 90.86 |
|  | bta-miR-2285av | | 176.86 | 90.86 |
|  | bta-miR-141 | | 136.86 | 55.14 |
|  | bta-miR-196a | | 67.86 | 118.00 |
|  | bta-miR-3432a | | 65.00 | 89.29 |
|  | bta-miR-3432a | | 63.71 | 87.14 |
|  | bta-miR-196a | | 51.86 | 88.00 |
|  | bta-miR-16b | | 97.57 | 41.00 |
|  | bta-miR-93 | | 82.00 | 44.00 |
|  | bta-miR-92a | | 94.86 | 25.29 |
|  | bta-miR-660 | | 83.43 | 34.29 |
|  | bta-miR-92a | | 87.29 | 24.00 |
|  | bta-miR-193a-5p | | 42.43 | 68.43 |
|  | bta-miR-99a-5p | | 59.00 | 32.57 |
|  | bta-miR-103 | | 51.57 | 38.57 |
|  | bta-miR-103 | | 51.57 | 37.57 |
|  | bta-miR-21-5p | | 65.57 | 20.29 |
|  | bta-miR-6529a | | 60.43 | 20.86 |
|  | bta-miR-30e-5p | | 41.00 | 37.86 |
|  | bta-miR-2284ab | | 28.71 | 23.29 |
|  | bta-miR-2285by | | 28.71 | 23.29 |
|  | bta-miR-24-3p | | 32.29 | 19.00 |
|  | bta-miR-24-3p | | 31.71 | 19.00 |
|  | bta-miR-11987 | | 21.57 | 28.86 |
|  | bta-miR-155 | | 26.29 | 23.29 |
|  | bta-miR-185 | | 32.00 | 16.29 |
|  | bta-miR-1 | | 41.71 | 5.71 |
|  | bta-miR-1 | | 41.71 | 5.71 |
|  | bta-miR-200b | | 38.43 | 9.00 |
|  | bta-miR-223 | | 37.86 | 8.86 |
|  | bta-miR-29a | | 30.29 | 15.86 |
|  | bta-miR-181a | | 24.14 | 21.00 |
|  | bta-miR-181a | | 24.14 | 21.00 |
|  | bta-miR-532 | | 22.43 | 18.14 |
|  | bta-miR-877 | | 19.14 | 21.14 |
|  | bta-miR-200a | | 25.29 | 13.00 |
|  | bta-miR-652 | | 19.57 | 17.71 |
|  | bta-miR-760-3p | | 13.29 | 19.00 |
|  | bta-miR-125a | | 20.00 | 10.86 |
|  | bta-miR-2887 | | 7.57 | 20.71 |
|  | bta-miR-2887 | | 7.57 | 20.71 |
|  | bta-miR-296-3p | | 18.00 | 10.00 |
|  | bta-miR-23a | | 24.14 | 3.71 |
|  | bta-miR-1307 | | 17.71 | 9.29 |
|  | bta-miR-339a | | 22.71 | 2.29 |
|  | bta-miR-339b | | 22.71 | 2.29 |
|  | bta-miR-29b | | 17.57 | 6.71 |
|  | bta-miR-29b | | 17.57 | 6.71 |
|  | bta-miR-186 | | 17.29 | 6.43 |
|  | bta-miR-181b | | 10.29 | 12.86 |
|  | bta-miR-181b | | 10.14 | 12.86 |
|  | bta-miR-29c | | 18.86 | 4.14 |
|  | bta-miR-151-3p | | 13.14 | 9.29 |
|  | bta-miR-11971 | | 12.14 | 9.57 |
|  | bta-miR-339b | | 18.57 | 2.29 |
|  | bta-miR-125b | | 12.71 | 7.86 |
|  | bta-miR-125b | | 12.71 | 7.86 |
|  | bta-miR-22-3p | | 12.29 | 5.00 |
|  | bta-miR-30f | | 14.86 | 1.43 |
|  | bta-miR-425-5p | | 10.57 | 5.57 |
|  | bta-miR-6525 | | 6.29 | 8.29 |
|  | bta-miR-130b | | 6.71 | 7.43 |
|  | bta-miR-11975 | | 9.00 | 5.00 |
|  | bta-miR-11976 | | 9.00 | 5.00 |
|  | bta-miR-11975 | | 9.00 | 5.00 |
|  | bta-miR-11976 | | 9.00 | 5.00 |
|  | bta-miR-11986b | | 9.57 | 3.86 |
|  | bta-miR-10174-3p | | 10.57 | 2.57 |
|  | bta-miR-23b-3p | | 10.57 | 2.57 |
|  | bta-miR-30b-3p | | 2.43 | 10.43 |
|  | bta-miR-2284w | | 8.71 | 3.71 |
|  | bta-miR-182 | | 7.57 | 4.71 |
|  | bta-miR-744 | | 6.86 | 5.29 |
|  | bta-miR-107 | | 9.57 | 2.29 |
|  | bta-miR-421 | | 8.14 | 3.71 |
|  | bta-miR-30b-5p | | 8.57 | 3.14 |
|  | bta-miR-34a | | 4.86 | 6.71 |
|  | bta-miR-23b-3p | | 8.43 | 2.43 |
|  | bta-miR-361 | | 8.00 | 2.57 |
|  | bta-miR-1839 | | 6.57 | 3.86 |
|  | bta-miR-148a | | 5.86 | 4.43 |
|  | bta-miR-221 | | 7.14 | 2.86 |
|  | bta-miR-342 | | 8.14 | 1.86 |
|  | bta-miR-15b | | 8.14 | 1.43 |
|  | bta-miR-143 | | 3.14 | 6.00 |
|  | bta-miR-429 | | 5.14 | 4.00 |
|  | bta-miR-20a | | 6.29 | 2.29 |
|  | bta-miR-2284y | | 5.14 | 3.43 |
|  | bta-miR-2285av | | 5.14 | 3.43 |
|  | bta-miR-2284y | | 5.14 | 3.43 |
|  | bta-miR-2285av | | 5.14 | 3.43 |
|  | bta-miR-2284y | | 5.14 | 3.43 |
|  | bta-miR-2285av | | 5.14 | 3.43 |
|  | bta-miR-2284h-5p | | 5.00 | 3.57 |
|  | bta-miR-2284y | | 5.00 | 3.57 |
|  | bta-miR-2285av | | 5.00 | 3.57 |
|  | bta-miR-2284y | | 5.14 | 3.43 |
|  | bta-miR-2285av | | 5.14 | 3.43 |
|  | bta-miR-2284y | | 5.14 | 3.43 |
|  | bta-miR-2285av | | 5.14 | 3.43 |
|  | bta-miR-2284y | | 5.14 | 3.43 |
|  | bta-miR-2285av | | 5.14 | 3.43 |
|  | bta-miR-99b | | 4.14 | 4.43 |
|  | bta-miR-31 | | 3.71 | 4.29 |
|  | bta-miR-500 | | 4.14 | 3.86 |
|  | bta-miR-30c | | 6.86 | 0.71 |
|  | bta-miR-2285av | | 4.00 | 3.29 |
|  | bta-miR-2448-3p | | 4.86 | 2.43 |
|  | bta-miR-25 | | 5.43 | 1.43 |
|  | bta-miR-423-3p | | 5.43 | 1.43 |
|  | bta-miR-885 | | 3.14 | 3.57 |
|  | bta-miR-11975 | | 6.43 | 0.14 |
|  | bta-miR-671 | | 4.14 | 2.43 |
|  | bta-miR-2285t | | 4.86 | 1.57 |
|  | bta-miR-28 | | 2.14 | 4.14 |
|  | bta-miR-345-3p | | 5.00 | 1.14 |
|  | bta-miR-425-3p | | 4.14 | 2.00 |
|  | bta-miR-484 | | 4.29 | 1.71 |
|  | bta-miR-365-3p | | 5.57 | 0.00 |
|  | bta-miR-365-3p | | 5.57 | 0.00 |
|  | bta-miR-378 | | 3.43 | 2.14 |
|  | bta-miR-335 | | 2.29 | 2.86 |
|  | bta-miR-2299-3p | | 3.00 | 1.71 |
|  | bta-miR-142-5p | | 4.57 | 0.00 |
|  | bta-miR-664b | | 3.43 | 1.14 |
|  | bta-miR-1306 | | 3.14 | 1.14 |
|  | bta-miR-7857-5p | | 2.00 | 2.29 |
|  | bta-miR-11981 | | 2.86 | 1.29 |
|  | bta-miR-7857-5p | | 1.86 | 2.29 |
|  | bta-miR-378 | | 2.29 | 1.71 |
|  | bta-miR-1343-5p | | 2.29 | 1.43 |
|  | bta-miR-2419-5p | | 2.57 | 1.00 |
|  | bta-miR-1260b | | 2.00 | 1.43 |
|  | bta-miR-2285by | | 1.29 | 2.14 |
|  | bta-miR-142-3p | | 2.57 | 0.71 |
|  | bta-miR-29d-5p | | 2.86 | 0.43 |
|  | bta-miR-29d-5p | | 2.86 | 0.43 |
|  | bta-miR-27a-3p | | 2.86 | 0.29 |
|  | bta-miR-362-5p | | 1.29 | 1.86 |
|  | bta-miR-12030 | | 2.57 | 0.43 |
|  | bta-miR-92b | | 0.43 | 2.57 |
|  | bta-miR-140 | | 2.86 | 0.00 |
|  | bta-miR-149-5p | | 1.57 | 1.29 |
|  | bta-miR-10a | | 1.29 | 1.43 |
|  | bta-miR-324 | | 1.43 | 1.29 |
|  | bta-miR-1249 | | 2.00 | 0.57 |
|  | bta-miR-146b | | 2.14 | 0.43 |
|  | bta-miR-2285au | | 1.14 | 1.43 |
|  | bta-miR-2285au | | 1.14 | 1.43 |
|  | bta-miR-2285au | | 1.14 | 1.43 |
|  | bta-miR-147 | | 1.29 | 1.14 |
|  | bta-miR-3432b | | 1.14 | 1.29 |
|  | bta-miR-12034 | | 2.29 | 0.00 |
|  | bta-miR-2387 | | 1.29 | 1.00 |
|  | bta-miR-331-3p | | 1.57 | 0.71 |
|  | bta-miR-6120-3p | | 2.29 | 0.00 |
|  | bta-miR-6517 | | 1.14 | 1.14 |
|  | bta-miR-15a | | 1.43 | 0.71 |
|  | bta-miR-183 | | 0.71 | 1.43 |
|  | bta-miR-22-5p | | 1.43 | 0.71 |
|  | bta-miR-27b | | 1.00 | 1.14 |
|  | bta-miR-363 | | 2.00 | 0.14 |
|  | bta-miR-374a | | 0.57 | 1.57 |
|  | bta-miR-769 | | 1.29 | 0.86 |
|  | bta-miR-128 | | 1.43 | 0.57 |
|  | bta-miR-17-5p | | 1.00 | 1.00 |
|  | bta-miR-328 | | 0.71 | 1.29 |
|  | bta-miR-6523a | | 0.71 | 1.14 |
|  | bta-miR-10179-5p | | 0.29 | 1.43 |
|  | bta-miR-128 | | 1.43 | 0.14 |
|  | bta-miR-2346 | | 1.00 | 0.57 |
|  | bta-miR-2388-3p | | 0.00 | 1.57 |
|  | bta-miR-126-3p | | 1.00 | 0.43 |
|  | bta-miR-17-3p | | 1.29 | 0.14 |
|  | bta-miR-1949 | | 0.86 | 0.57 |
|  | bta-miR-12057 | | 0.43 | 0.86 |
|  | bta-miR-122 | | 0.00 | 1.29 |
|  | bta-miR-130a | | 0.14 | 1.14 |
|  | bta-miR-210 | | 1.00 | 0.29 |
|  | bta-miR-2285aj-5p | | 0.57 | 0.71 |
|  | bta-miR-374b | | 1.29 | 0.00 |
|  | bta-miR-2284aa | | 0.71 | 0.43 |
|  | bta-miR-2284aa | | 0.71 | 0.43 |
|  | bta-miR-2372 | | 1.14 | 0.00 |
|  | bta-miR-2443 | | 0.43 | 0.71 |
|  | bta-miR-326 | | 0.86 | 0.29 |
|  | bta-miR-150 | | 0.43 | 0.57 |
|  | bta-miR-206 | | 1.00 | 0.00 |
|  | bta-miR-2284aa | | 0.57 | 0.43 |
|  | bta-miR-2284aa | | 0.57 | 0.43 |
|  | bta-miR-2285k | | 1.00 | 0.00 |
|  | bta-miR-2285k | | 1.00 | 0.00 |
|  | bta-miR-2285k | | 1.00 | 0.00 |
|  | bta-miR-6522 | | 1.00 | 0.00 |
|  | bta-miR-10176-5p | | 0.00 | 0.86 |
|  | bta-miR-11972 | | 0.86 | 0.00 |
|  | bta-miR-11973 | | 0.86 | 0.00 |
|  | bta-miR-139 | | 0.86 | 0.00 |
|  | bta-miR-18a | | 0.86 | 0.00 |
|  | bta-miR-193a-3p | | 0.86 | 0.00 |
|  | bta-miR-197 | | 0.86 | 0.00 |
|  | bta-miR-2284j | | 0.86 | 0.00 |
|  | bta-miR-2458 | | 0.00 | 0.86 |
|  | bta-miR-296-5p | | 0.43 | 0.43 |
|  | bta-miR-378b | | 0.86 | 0.00 |
|  | bta-miR-382 | | 0.00 | 0.86 |
|  | bta-miR-6518 | | 0.86 | 0.00 |
|  | bta-miR-100 | | 0.57 | 0.14 |
|  | bta-miR-11988 | | 0.71 | 0.00 |
|  | bta-miR-12061 | | 0.71 | 0.00 |
|  | bta-miR-20b | | 0.14 | 0.57 |
|  | bta-miR-2285ak-5p | | 0.57 | 0.14 |
|  | bta-miR-2336 | | 0.14 | 0.57 |
|  | bta-miR-3141 | | 0.00 | 0.71 |
|  | bta-miR-486 | | 0.57 | 0.14 |
|  | bta-miR-199a-3p | | 0.57 | 0.00 |
|  | bta-miR-199c | | 0.57 | 0.00 |
|  | bta-miR-199a-3p | | 0.57 | 0.00 |
|  | bta-miR-199c | | 0.57 | 0.00 |
|  | bta-miR-199a-3p | | 0.57 | 0.00 |
|  | bta-miR-199c | | 0.57 | 0.00 |
|  | bta-miR-224 | | 0.00 | 0.57 |
|  | bta-miR-2284v | | 0.14 | 0.43 |
|  | bta-miR-2284aa | | 0.57 | 0.00 |
|  | bta-miR-2284z | | 0.57 | 0.00 |
|  | bta-miR-2284aa | | 0.57 | 0.00 |
|  | bta-miR-2284z | | 0.57 | 0.00 |
|  | bta-miR-2284aa | | 0.57 | 0.00 |
|  | bta-miR-2284z | | 0.57 | 0.00 |
|  | bta-miR-2284aa | | 0.57 | 0.00 |
|  | bta-miR-2284z | | 0.57 | 0.00 |
|  | bta-miR-2284aa | | 0.57 | 0.00 |
|  | bta-miR-2284z | | 0.57 | 0.00 |
|  | bta-miR-2284aa | | 0.57 | 0.00 |
|  | bta-miR-2284z | | 0.57 | 0.00 |
|  | bta-miR-2284aa | | 0.57 | 0.00 |
|  | bta-miR-2284z | | 0.57 | 0.00 |
|  | bta-miR-2426 | | 0.57 | 0.00 |
|  | bta-miR-499 | | 0.00 | 0.57 |
|  | bta-miR-6715 | | 0.57 | 0.00 |
|  | bta-miR-10167-3p | | 0.43 | 0.00 |
|  | bta-miR-106b | | 0.14 | 0.29 |
|  | bta-miR-12056 | | 0.00 | 0.43 |
|  | bta-miR-181d | | 0.29 | 0.14 |
|  | bta-miR-196b | | 0.14 | 0.29 |
|  | bta-miR-2285k | | 0.43 | 0.00 |
|  | bta-miR-2285k | | 0.43 | 0.00 |
|  | bta-miR-411a | | 0.43 | 0.00 |
|  | bta-miR-424-5p | | 0.43 | 0.00 |
|  | bta-miR-432 | | 0.00 | 0.43 |
|  | bta-miR-6520 | | 0.43 | 0.00 |
|  | bta-miR-95 | | 0.43 | 0.00 |
|  | bta-miR-99a-3p | | 0.14 | 0.29 |
|  | bta-miR-10225b | | 0.29 | 0.00 |
|  | bta-miR-11994 | | 0.00 | 0.29 |
|  | bta-miR-133a | | 0.14 | 0.14 |
|  | bta-miR-133a | | 0.14 | 0.14 |
|  | bta-miR-145 | | 0.00 | 0.29 |
|  | bta-miR-187 | | 0.29 | 0.00 |
|  | bta-miR-18b | | 0.00 | 0.29 |
|  | bta-miR-194 | | 0.29 | 0.00 |
|  | bta-miR-194 | | 0.29 | 0.00 |
|  | bta-miR-19b | | 0.29 | 0.00 |
|  | bta-miR-19b | | 0.29 | 0.00 |
|  | bta-miR-2284k | | 0.14 | 0.14 |
|  | bta-miR-2332 | | 0.14 | 0.14 |
|  | bta-miR-2389 | | 0.29 | 0.00 |
|  | bta-miR-2410 | | 0.29 | 0.00 |
|  | bta-miR-27a-5p | | 0.14 | 0.14 |
|  | bta-miR-6527 | | 0.00 | 0.29 |
|  | bta-miR-7859 | | 0.14 | 0.14 |
|  | bta-let-7a-3p | | 0.14 | 0.00 |
|  | bta-let-7a-3p | | 0.14 | 0.00 |
|  | bta-miR-11978 | | 0.00 | 0.14 |
|  | bta-miR-11985 | | 0.14 | 0.00 |
|  | bta-miR-12023 | | 0.14 | 0.00 |
|  | bta-miR-12064 | | 0.14 | 0.00 |
|  | bta-miR-124a | | 0.14 | 0.00 |
|  | bta-miR-124b | | 0.14 | 0.00 |
|  | bta-miR-124a | | 0.14 | 0.00 |
|  | bta-miR-124b | | 0.14 | 0.00 |
|  | bta-miR-124a | | 0.14 | 0.00 |
|  | bta-miR-124b | | 0.14 | 0.00 |
|  | bta-miR-132 | | 0.14 | 0.00 |
|  | bta-miR-1388-3p | | 0.14 | 0.00 |
|  | bta-miR-190a | | 0.14 | 0.00 |
|  | bta-miR-192 | | 0.14 | 0.00 |
|  | bta-miR-194b | | 0.14 | 0.00 |
|  | bta-miR-194b-3p | | 0.14 | 0.00 |
|  | bta-miR-194b | | 0.14 | 0.00 |
|  | bta-miR-215 | | 0.00 | 0.14 |
|  | bta-miR-2284a | | 0.14 | 0.00 |
|  | bta-miR-2284d | | 0.14 | 0.00 |
|  | bta-miR-2284h-5p | | 0.14 | 0.00 |
|  | bta-miR-2284m | | 0.00 | 0.14 |
|  | bta-miR-2284p | | 0.14 | 0.00 |
|  | bta-miR-2285aa | | 0.14 | 0.00 |
|  | bta-miR-2285bn | | 0.14 | 0.00 |
|  | bta-miR-2285bo | | 0.00 | 0.14 |
|  | bta-miR-2285bs | | 0.14 | 0.00 |
|  | bta-miR-2285bt | | 0.14 | 0.00 |
|  | bta-miR-2285cj | | 0.14 | 0.00 |
|  | bta-miR-2285cy | | 0.14 | 0.00 |
|  | bta-miR-2285da | | 0.14 | 0.00 |
|  | bta-miR-2355-5p | | 0.14 | 0.00 |
|  | bta-miR-2403 | | 0.14 | 0.00 |
|  | bta-miR-2411-5p | | 0.14 | 0.00 |
|  | bta-miR-29d-3p | | 0.14 | 0.00 |
|  | bta-miR-340 | | 0.14 | 0.00 |
|  | bta-miR-345-5p | | 0.14 | 0.00 |
|  | bta-miR-365-5p | | 0.14 | 0.00 |
|  | bta-miR-378c | | 0.14 | 0.00 |
|  | bta-miR-491 | | 0.14 | 0.00 |
|  | bta-miR-6123 | | 0.14 | 0.00 |
|  | bta-miR-628 | | 0.14 | 0.00 |
